# A Comparative Evaluation of Efficacy of Different Obturation Techniques used in Root Canal Treatment of Anterior Teeth: An *in vitro *Study

**DOI:** 10.5005/jp-journals-10005-1224

**Published:** 2014-04-26

**Authors:** Firoza Samadi, JN Jaiswal, Sonali Saha, Nishita Garg, Swati Chowdhary, Fahad Samadi, Vandana Pandey Tripathi

**Affiliations:** Professor and Head, Department of Pedodontics and Preventive Dentistry Sardar Patel Post Graduate Institute of Dental and Medical Sciences, Lucknow, Uttar Pradesh, India; Professor and Director, Department of Pedodontics and Preventive Dentistry Sardar Patel Post Graduate Institute of Dental and Medical Sciences, Lucknow, Uttar Pradesh, India; Senior Lecturer, Department of Pedodontics and Preventive Dentistry Sardar Patel Post Graduate Institute of Dental and Medical Sciences, Lucknow, Uttar Pradesh, India; Senior Lecturer, Department of Pedodontics and Preventive Dentistry Sardar Patel Post Graduate Institute of Dental and Medical Sciences, Lucknow, Uttar Pradesh, India; Senior Lecturer, Department of Pedodontics and Preventive Dentistry, Manav Rachana Dental College, Lucknow, Uttar Pradesh, India; Senior Lecturer, Department of Oral and Maxillofacial Pathology, Sardar Patel Post Graduate Institute of Dental and Medical Sciences Lucknow, Uttar Pradesh, India; Postgraduate, Department of Pedodontics and Preventive Dentistry Sardar Patel Post Graduate Institute of Dental and Medical Sciences, Lucknow, Uttar Pradesh, India

**Keywords:** Gutta-percha filled area, Thermafil, Warm vertical, Cold lateral condensation

## Abstract

**Aim: **This study was undertaken to evaluate the percentage of gutta-percha-filled area (PGFA) using microscopic analysis of the cross-sections in the apical third of root canals when filled either with Thermafil technique, Warm Vertical Condensation technique and Cold Lateral Condensation technique without using sealers.

**Materials and methods: **Sixty single rooted extracted per-manent teeth were collected. After crown amputation, the teeth were randomly divided into three experimental groups of 20 specimens each. Group I–Thermafil obturation technique, group II–warm vertical condensation obturation technique and group III–cold lateral condensation obturation technique. Obturation was performed by specific techniques without using sealers. After obturation, the teeth were cross-sectioned horizontally at 2 to 3 mm from apex with the help of double sided diamond disk. Sections were digitally photographed and measured under Stereomicroscope at magnification of 50×.

Using a KS 100 imaging system the area of canals and the gutta-percha was recorded, also the percentage of gutta-percha filled area (PGFA) was calculated. The observations thus ob-tained were subjected to statistical analysis using ANOVA and student ‘t’ test.

**Results: **Maximum group difference was observed between groups I and III (3.558 ± 0.138) while minimum difference was observed between groups I and II (1.223 ± 0.137). Thus, all the between group differences were statistically significant.

**Conclusion: **This study supports the belief that the Thermafil Obturation technique produces significantly higher percentage of gutta-percha filled area (PGFA) than the warm vertical condensation technique or cold lateral condensation technique.

**How to cite this article: **Samadi F, Jaiswal JN, Saha S, Garg N, Chowdhary S, Samadi F, Tripathi VP. A Comparative Evaluation of Efficacy of Different Obturation Techniques used in Root Canal Treatment of Anterior Teeth: An *in vitro *Study. Int J Clin Pediatr Dent 2014;7(1):1-5.

## INTRODUCTION

Successful endodontic therapy is critically dependent on the thorough removal of microorganisms and their by-products through mechanical root canal instrumentation, antibacterial irrigation and adequate filling of the root canal space.^1^ The goal of root canal filling is to completely obliterate the canal space with a stable, nontoxic material and at the same time creating a hermetic seal to prevent the movement of tissue fluids, bacteria or bacterial by-products through the filled canal.^2^ Obturation provides a seal that prevents reinfection of the canal and subsequent leakage into the periradicular tissues.^3^

Although there are many techniques for obturation of root canals, but still search is on for better techniques, as cold lateral condensation (CLC) technique, the most frequently used technique and the standard with which all other techni-ques are compared, results in creation of voids, spreader tracts and lack of surface adaptation to canal walls.^4^

In recent years, a number of plasticized gutta-percha techniques have been introduced that have purported to seal the root canal better, like Warm Vertical Compaction technique (WVC) and Thermafil obturation technique which incorporate the use of thermal or frictional heat to plasticize the gutta-percha, allowing for better adaptation to canal walls, higher degree of homogeneity and provide optimum apical and coronal sealing when compared to lateral condensation.^4,5^ The WVC technique takes advantage of excellent gutta-percha filling as close as possible to the apex. The Thermafil obturation technique produces higher radiopacity, excellent viscosity and fluidity and produces a high degree homogenous mass of gutta-percha in the canal unlike lateral condensation.^6^

Hence, the present study was undertaken to evaluate the percentage of gutta-percha-filled area (PGFA) using microscopic analysis of the cross-sections in the apical third of root canals when filled either with Thermafil technique, WVC technique and CLC technique.

## MATERIALS AND METHODS

**Fig. 1:**
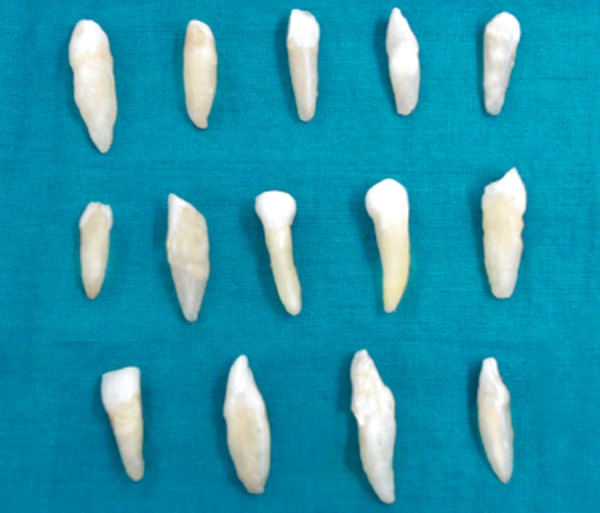
Freshly extracted single-rooted permanent teeth

Sixty single rooted extracted permanent teeth with single straight canals were used for this study (Fig. 1). Teeth were collected and cleaned then stored in sodium hypochlorite solution (3%) for 2 weeks. The pulp chambers of all the teeth were opened and number 15 K-file was introduced in the access cavity to establish the patency of the canal. The working length of each tooth was measured with the aid of radio visiography. Biomechanical preparation was carried out using step back technique. All the teeth were instrumented at the working length with number 50 K-file.

The teeth were randomly divided into three groups accordingly to the filling technique used (n = 20), CLC tech-nique, WLC technique and Thermafil technique. Obturation was performed by specific techniques without using sealers.

### Group I: Cold lateral Condensation Obturation Technique

A standardized 50 number gutta-percha master cone was fitted in the root canal at the working length and checked for tug-back criteria. Cold lateral condensation was done using standardized finger spreaders. The finger spreader was inserted between the master cone and the canal wall to within 1 mm of the working length. Cold lateral condensation was performed using accessory gutta-percha points until the canals were completely filled. The filling was judged to be completed when size 15 finger spreader could not penetrate beyond the coronal third of the canal. Excess gutta-percha was removed with the hot instrument.

### Group II: Thermafil Obturation Technique (Fig. 2)

A size 50 Thermafil verifier was used to check the size of the canal and thus, the correct Thermafil obturator. The Thermafil plastic obturator (Dentsply) was heated in a Thermaprep Oven (Dentsply) for 30 seconds according to the manufacturer's instructions. Firm apical pressure was used to insert the Thermafil obturator to the working length. A round diamond bur in a turbine handpiece was used to cut the plastic shaft at 1 to 2 mm within the access cavity and the excess gutta-percha was removed with an instrument.

### Group III: Warm Vertical Condensation Obturation Technique

For this technique, various hand held pluggers were used. Three pluggers whose diameter were just slightly less than that of canal preparation at any prior level were selected for working on the coronal, middle and apical 1/3rd of canal. After master cone insertion, a spreader was heated to 60°C in a glass bead sterilizer and allowed to plunge 3 to 4 mm into the apical most extent of gutta-percha and was allowed to remain there till it began to cool. Then it was removed and largest prefitted plugger was used to vertically pack gutta-percha mass apically. This procedure was repeated till the canal was fully filled.

### Sectioning and Image Analysis

After obturation, the teeth were cross-sectioned horizon-tally at 2 to 3 mm from apex with the help of double-sided diamond disk. Color studies of sections were taken using a Stereomicroscope at magnification of 50× (Fig. 3). The slides were scanned as tagged image file format (TIFFA) images. Using a KS 100 imaging system the area of canals and the gutta-percha was recorded and the PGFA was calculated. The measurements were repeated randomly in at least two sec-tions per group to assure reproducibility of measurements.

**Fig. 2:**
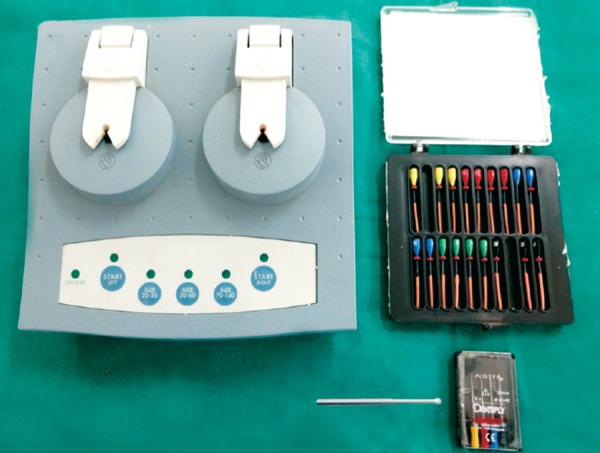
Instruments and materials used for Thermafil obturation technique

**Fig. 3:**
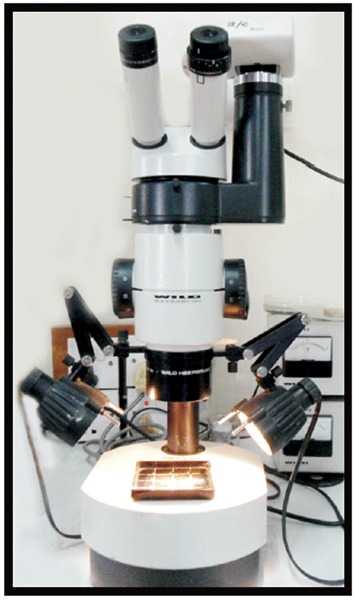
Stereomicroscope

**Fig. 4:**
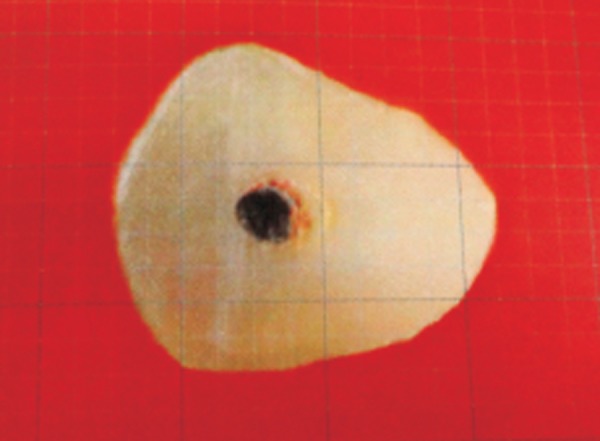
Void calculated on grid scale (Thermafil)

**Fig. 5:**
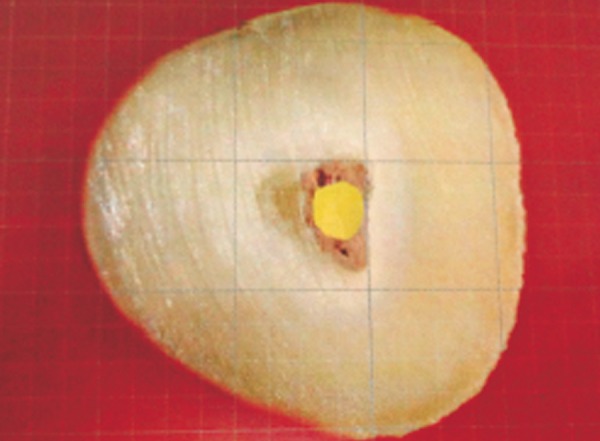
Void calculated on grid scale (warm vertical condensation)

**Fig. 6:**
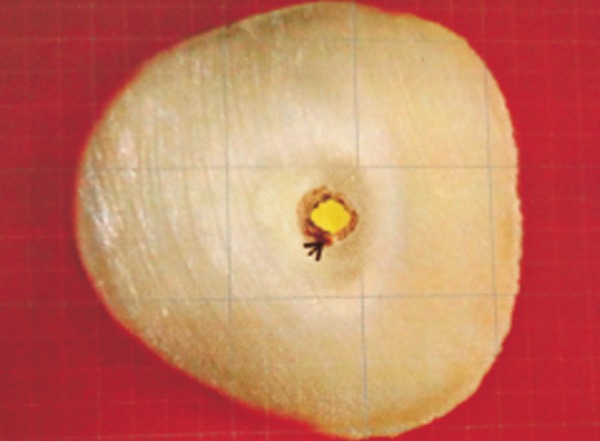
Void calculated on grid scale (cold lateral condensation)

### Statistical Analysis

The observations thus obtained were subjected to statistical analysis using analysis of variance (ANOVA), Post hoc and Student ‘t’ test were performed to know the effect of each variable and to reveal the statistical significance. The confidence level of study was proposed to be 95% hence p-value < 0.05 has been considered significant, p-value < 0.01 has been considered highly significant and p-value < 0.001 has been considered very highly significant.

## RESULTS

Table 1 shows the mean percentage of gutta-percha filled area in Thermafil technique, WVC technique and CLC technique at apical third of root canals. It was observed that the mean PGFA was maximum in group I (Thermafil tech-nique) followed by group II (WVC) while group III (CLC techniqueh) revealed the minimum percentage (Figs 4 to 6).

Table 2 shows analysis of variance (ANOVA) of PGFA in different study groups. Result reveals highly significant intergroup difference (p < 0.001).

It was observed that there was no overlapping in inter-quartile ranges of any of the three study groups.

Table 3 shows group comparison of percentage of gutta-percha filled area in different study groups. Result shows that the maximum group difference was observed between groups I and III (3.558 ± 0.138) while minimum difference was observed between groups I and II (1.223 ± 0.137).

Group I (Thermafil) had significantly higher mean value as compared to the two study groups (groups II and III) and group II (WVC) had significantly higher mean value as compared to group III (CLC). Thus, the differences among all the groups were statistically significant.

**Table 1:** **Table 1: **Mean percentage of gutta-percha filled area in different study groups

*S.no.*		*Group*		*No. of samples*		*Mean (%)*		*SD*			
1.		I		20		98.79		0.21			
2.		II		20		97.57		0.22			
3.		III		20		95.24		0.69			

**Table 2:** **Table 2: **Analysis of variance of percentage of gutta-percha filled area in different groups

		*Sum of squares*		*Df*		*Mean square*		*“F”*		*Significance (p)*	
Between groups		130.67		2		65.334		344.702		<0.001			
Within groups		10.80		57		0.190		–		–			
Total		141.47		59		–		–		–	

**Table 3:** **Table 3: **Between group comparisons of percentage of gutta-percha filled area in different groups

*S. no.*		*Comparison*		*Mean difference*		*SE of diff.*		*p-value*			
1.		Group I *vs *group II		1.223		0.138		<0.001			
2.		Group I *vs *group III		3.558		0.138		<0.001			
3.		Group II *vs *group III		2.334		0.138		<0.001			

## DISCUSSION

The main purpose of endodontic treatment is to clean, shape and fll the root canal space thoroughly and prevent any interchange between the oral cavity, the root canal system and periradicular tissues, providing a barrier to reinfection. The success of endodontic treatment depends on adequate mechanical debridement of root canal and quality obturation with biocompatible material.^5^

Fogel (1995) and Wu et al (1998) proposed that the quality of root canal filling at the apical third is important because after post space preparation only the apical root filling of 3 or 4 mm in length is left and the cemented post may leak.^6,7^ According to various investigators [Senia et al (1971), Coffae and Brilliant (1975), Littman (1977)]^8-10^ the apical root canal has been found to be less clean than the middle and coronal portions of the root canal suggesting that bacteria remain in the apical canal. In the present study no sealer was used although generally recommended during conventional root canal filling procedure. Peters (1986) and Georgopoulou et al (1995) stated that sealer shrinks upon setting while others are susceptible to dissolution in contact with tissue fluids leading to increase in leakage along the root filling over time.^11,12^ If sealers had been used there would have been variations regarding the width of root canal, the depth of the heat application and also the size of the sealer filled canal area. Although including sealer may facilitate gutta-percha movement, the reasons to leave sealers out were more important.

In the present study maxillary anterior teeth were used in attempts to avoid the presence of isthmus or fattening area, commonly found in molars and mandibular incisors.

The prepared teeth were randomly divided and obturated in following three groups (n = 20 per group) namely Thermafil technique (group I), WVC technique (group II) and CLC technique (group III).

Cold lateral condensation technique is a common method for obturation. According to Gordon et al (2005) and Xu et al (2007) CLC technique serves as the gold-standard against which new techniques are compared. In the present study, though the density of gutta-percha was found to be relatively good, but obvious voids and spreader tracts were apparent in the cross-sections.^13,14^

The other two groups (WVC technique and Thermafil technique) with PGFA greater than that of CLC utilized some form of heat to plasticize the gutta-percha.

Warm vertical condensation technique should result in the plasticizing of the gutta-percha apical to the heat carrier. It can be concluded that after WVC the quality of the adaptation of gutta-percha to the wall of the apical root canal varies and the sufficient amount of gutta-percha present in the apical canal and sufficient heating are essential in achieving a good adaptation in canals of widely varying diameter. Venturi and Breschi (2004) reported that multiple heating has shown to produce high levels of gutta-percha shrinkage.^15^ It is possible that heated gutta-percha simply fows and expands around the pluggers instead of being forced in to the irregularities. These could be the reasons why WVC technique showed less percentage of gutta-percha filled area compared to the Thermafil technique.

The group filled by the Thermafil system at the apical third (2-3 mm from the apical foramen) revealed the best results. Thermafil samples presented a more homogeneous mass which included gutta-percha and the plastic carrier. Thermafil obturators are flexible plastic carrier coated with alpha phase gutta-percha while extruder of element obtu-rating unit contains beta phase gutta-percha. Alpha phase gut-tapercha has low melting temperature and good adhesiveness whereas beta phase gutta-percha has high melting points and no properties of adhesion. Hence the potential for shrinkage of thermoplasticized gutta-percha as used in Thermafil should be lesser than element obturation unit it has excellent viscosity, fluidity and enhanced adherence replicating the root better and have fewer voids.

The thermoplastic gutta-percha techniques are simpler than the lateral condensation technique which means the operator is less subjected to fatigue, but such consideration must be subordinate to the primary goal of achieving the best prognosis for the patients.

After obturation, each sample was sectioned horizontally at 2 to 3 mm from the apical foramen using low-speed saw (Isomet, Buhler Ltd, Lake Bluff, NY, USA) with a diamond disk (125 × 0.35 × 12.7 mm – 330c) and continuous water irrigation in order to prevent overheating. The specimens were polished before they were examined under stereomicro-scope.

This study was designed to quantify the gutta-percha component on percentage basis in order to provide a measure of quality. As observed in the present study, the mean PGFA was found to be maximum in group I (Thermafil technique) followed by group II (WVC) and group III (CLC technique).

## CONCLUSION

On the basis of the results and statistical analysis, it can be concluded that:

1. Cold lateral obturation technique resulted in definite voids and gaps between the gutta-percha and canal interface at apical third of root canals.
2. Warm vertical condensation technique produce a homogenous mass of gutta-percha with reduced voids and increased adaptation as compared to cold lateral obturation technique.
3. Thermafil obturation technique exhibited least voids and gaps compared to other tested techniques.
4. Obturation of root canals with alpha-phase gutta-percha on a plastic core-carrier, Thermafil, resulted in a more dense and well adapted root canal fll at the apical third in comparison to the cold lateral obturation technique and WVC technique.
5. None of the tested obturation techniques provided a gap-free or void-free root canal filling.
